# Schistosomiasis Control in Nigeria: Moving Round the Circle?

**DOI:** 10.5334/aogh.2930

**Published:** 2020-07-06

**Authors:** Oyetunde T. Oyeyemi

**Affiliations:** 1 Department of Biological Sciences, University of Medical Sciences, Ondo, NG; 2 Diagnosis and Therapy of Infectious Diseases and Cancer Laboratory, René Rachou Institute, Fundação Oswaldo Cruz, Belo Horizonte, Minas Gerais, BR

## Abstract

**Background:**

Schistosomiasis has continued to plague low-resource areas of the Nigerian population. Mass drug administration (MDA) has been the only adopted interventional program for decades. However, it appears this effort does not culminate in transmission and morbidity reduction.

**Purpose:**

To highlight the current situation of schistosomiasis in Nigeria, why MDA alone cannot achieve the expected result, identify research needs, and promotion of integrated control approach for schistosomiasis.

**Method:**

A viewpoint based on practices, research findings, and personal and professional experience in the field of schistosomiasis control.

**Conclusion:**

This viewpoint strongly advocates a commitment to the integrated control approach through the development of robust schistosomiasis control policy for the country. It stressed the need for research priorities in neglected areas of schistosomiasis that are germane for control of the disease. The government’s willpower to implement important recommendations from research outcomes is important to achieve success.

Nigeria, with 20 million people requiring schistosomiasis preventive chemotherapy, is ranked first amongst the countries of the world endemic for the disease [1]. While more efforts have been channeled to the control of schistosomiasis since the World Health Assembly (WHA) 2001 Resolution [2], transmission still appears unabated. With 74% mass drug administration (MDA) coverage index [3], Nigeria is about to meet up with the 75% minimum benchmark set for endemic countries. Despite this desirable feat, schistosomiasis appears to be a disease we shall still battle for another decade. Prevalence in school children can be as high as 70% [4], and in population once thought not to be at a high-risk group, 20% prevalence level has been observed [5].

Mass drug administration (MDA), which Nigeria solely relies on, is not sufficient to yield long-term control success. In Southwestern Nigeria, MDA in some areas was hampered with poor implementation owing to non-compliance to WHO guidelines on treatment. Visible hematuria was usually used to guide MDA is some instances, thus leaving out a large proportion of children with covert infection untreated. In some centres, suboptimal doses were administered as no weighing scale or praziquantel dose pole was used to determine the appropriate treatment dosage. The lack of synergy and partnership between research institutions and the Ministry of Health in Nigeria is also an important issue that needs to be addressed.

With decades of continuous MDA but without significant reduction in schistosomiasis morbidity level, transmission control, provision of good water supply, and understanding of the population social context that aids transmission may proffer a lasting solution. Nevertheless, the government of Nigeria and research institutions are not taking concerted efforts in this direction.

Nigeria can learn from countries such as China and Brazil with a strong national policy for the control of schistosomiasis. These countries, which have recorded tremendous success in schistosomiasis control, have adopted an integrated approach for decades. An effective and reliable diagnosis is paramount if Nigeria is to win the battle. It is important to state that three species of *Schistosoma* are currently recorded in Nigeria. Reports have shown a higher occurrence of *S. haematobium* in Southern Nigeria and *S. mansoni* in Northern Nigeria [6]. The third species *S. intercalatum* is hardly reported or mistaken for *S. haematobium*. Despite being the second most important parasitic infection after malaria in terms of the overall associated burdens, the country lacks reference to national laboratories for diagnosis, unlike the practice in Brazil and China. This, therefore, makes it difficult to monitor the current foci of transmission and proper evaluation of MDA. The impact of schistosomiasis in Nigeria does not measure up with the attention given to it. The purchase of ELISA diagnostic kits instead of local production of biological materials for immunodiagnostics is not cost-effective and not self-sustaining. So, few laboratories have the capacity for molecular diagnosis, and where present, schistosomiasis diagnosis is often not prioritized. This raises the concern of the true status of the disease in Nigeria, as a single diagnostic approach is no longer recommended for the monitoring of the disease progression in endemic areas. The Kato Katz technique for *S. mansoni* is hampered with the problem of poor sensitivity in light infection [7], and recent advances in schistosomiasis diagnosis have evolved into combination with other diagnostic approaches such as TF-test and ELISA.

Besides the usual prevalence studies, a lot is still required in the area of building capacity in training and research on schistosomiasis. Priorities should be placed on research on snail host and *Schistosoma* parasite relationship. The impact of freshwater snail hosts on the transmission of schistosomiasis cannot be overemphasized. In addition to the two main species known to transmit schistosomes in Nigeria, i.e., *Biomphalaria pfeifferi* and *Bulinus globosus*, new additions such as *Bulinus jousseaumei* and *B. camerunensis* with potential to transmit the *S. haematobium* were reported in Nigerian water bodies [8,9]. However, despite the rich research opportunities this presents to us, we are doing little to explore the schistosome-snail relationship. This research deficient in the field of medical malacology is not unconnected to a lack of resources for research engagement. At best, the few scientists in the field thrive through collaboration with partners in the developed countries. Nevertheless, much more is required. Priority should be given to studies on the identification of genes that determine snails’ susceptibility to infection and those that control other important physiological processes in the snails. This could help to identify some candidate genes that could be targeted by molluscicidal activities.

Transmission control has played a major role in China’s success against schistosomiasis [10]. Breaking of schistosome’s transmission cycle through the provision of clean and accessible water, proper waste disposal facilities, and snails’ eradication are germane in the integrated control approach. The provision of water and education could have been the game-changer in our control efforts. However, researchers are usually left with the only option of chemotherapy since the government lacks the willpower to implement recommendations on water supply in affected communities. Thus, chemotherapy, over the years, has not been sustainable, due to the problem of reinfection. Potable water and human waste disposal facilities should be provided within endemic communities and remote hotspots where human-water contacts and indiscriminate excretion or urination into water bodies occur. These efforts will yield the expected results only if accompanied by rigorous community health education. Experiences have shown that even in the presence of community water facilities, many still prefer to cool off and drink from these natural water bodies, which are the transmission foci of schistosomes. While we may currently lack the capacity to develop a tool that can detect water bodies infested by the parasites, eradication of snails by field application of niclosamide and black plastic film coverage of water surface, as done in China [10], can be sure ways of achieving effective schistosomiasis control.

It is high time we prioritized the treatment of all population groups and not only school children. Although school children are the most at-risk group, studies have shown other population strata to also be at high risk of infection. Pregnant women and preschoolers are two important groups our group identified as other high-risk groups [5,11,12,13]. Women of reproductive age are known to carry out laundry activities and fetch water for domestic purposes in these cercaria-infested water bodies while their infants or preschoolers often cool off in a shallow area of the water bodies under their mothers’ supervision, thus subjecting them to infection. While these groups and other adult populations are neglected in MDA programs, they become active sources of transmission to the treated population in resource-poor communities, thus rendering the area-wide infection prevalence and intensity to pre-control levels [12]. The morbid effects of non-treatment on pregnant women and preschoolers should inform the decision for their inclusion in treatment programs. Healthy growth may be compromised in preschoolers and chronic infection without treatment may worsen the long-term clinical picture of the disease in them [14]. In pregnant women, *Schistosoma* infection may result in adverse pregnancy outcomes such as low birth weight, abortion, stillbirth, and maternal death [15].

It is important to note that epidemiological data are still lacking in female genital schistosomiasis (FGS) despite its significant reproductive health impact. FGS affects at least 56 million women in endemic areas, causing considerable inequity by misdiagnosis and confusion with symptoms of sexually transmitted infections, social exclusion due to the impact on fertility, marital discord, depression, and sexual and reproductive morbidity related stigma for women and girls [16]. The infection may be so common in afflicted communities such that its clinical significance is downplayed and other sub-clinical morbidities are ignored. With current awareness on cervical cancer screening in Nigeria, integration of FGS screening into already existing cervical screening programs, especially in schistosomiasis endemic areas, could reduce its adverse reproductive health implications in affected women.

While it is certain we are yet to put the right mechanisms in place to control schistosomiasis, the current practice of ‘one size fits all’ in the control program can never achieve the desired outcomes. A robust national policy on schistosomiasis control is urgently needed in Nigeria. This policy should make adequate provision for other key areas often neglected in schistosomiasis control. Control efforts only skewed towards MDA as it is being currently practiced is not cost-effective due to recurrent infection and a repeated need for treatment. For successful implementation of MDA in schistosomiasis endemic areas, studies that address social context concerning the acceptability of MDA are recommended as none is currently available in Nigeria despite several years of MDA implementation in the country.
